# Translational Control of Alphavirus–Host Interactions: Implications in Viral Evolution, Tropism and Antiviral Response

**DOI:** 10.3390/v16020205

**Published:** 2024-01-30

**Authors:** Iván Ventoso, Juan José Berlanga, René Toribio, Irene Díaz-López

**Affiliations:** 1 Centro de Biología Molecular “Severo Ochoa” (CSIC-UAM) and Departamento de Biología Molecular, Universidad Autónoma de Madrid (UAM), 28049 Madrid, Spain; jberlanga@cbm.csic.es; 2 Centro de Biotecnología y Genómica de Plantas, Universidad Politécnica de Madrid-Instituto Nacional de Investigación y Tecnología Agraria y Alimentaria (UPM-INIA), 28049 Madrid, Spain; toribio.francisco@upm.es; 3 MRC Laboratory of Molecular Biology, Cambridge CB2 0QH, UK; idiaz@mrc-lmb.cam.ac.uk

**Keywords:** alphaviruses, translation initiation, eIF2α phosphorylation, RNA structure, antiviral response, interferon, evolution, host range

## Abstract

Alphaviruses can replicate in arthropods and in many vertebrate species including humankind, but only in vertebrate cells do infections with these viruses result in a strong inhibition of host translation and transcription. Translation shutoff by alphaviruses is a multifactorial process that involves both host- and virus-induced mechanisms, and some of them are not completely understood. Alphavirus genomes contain cis-acting elements (RNA structures and dinucleotide composition) and encode protein activities that promote the translational and transcriptional resistance to type I IFN-induced antiviral effectors. Among them, IFIT1, ZAP and PKR have played a relevant role in alphavirus evolution, since they have promoted the emergence of multiple viral evasion mechanisms at the translational level. In this review, we will discuss how the adaptations of alphaviruses to vertebrate hosts likely involved the acquisition of new features in viral mRNAs and proteins to overcome the effect of type I IFN.

## 1. Alphavirus Replication, Tropism and Interference with Host Gene Expression

**Figure 1:**
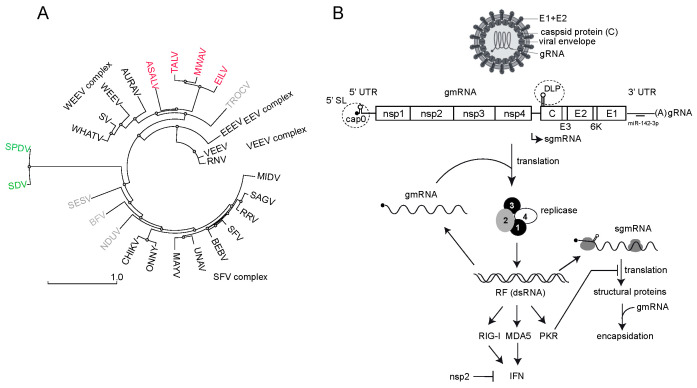
(**A**) Phylogenetic tree of some members of the *Alphavirus* genus. Structural polyprotein sequences (C-E3-E2-6K-E1) were aligned using Muscle, and the resulting distance-based phylogenetic tree was built using the tools available in the NGPhylogeny.fr suite. Some of the main complexes of the genus are shown. Insect-specific viruses close to the WEEV complex are in red. Aquatic alphaviruses infecting fish are in green. Members of other complexes are in light gray. (**B**) Schematic representation of alphavirus replication cycle including virion composition and genomic organization. The nonstructural (nsP1-3) and structural coding sequences (C-E3-E2-6K-E1) are indicated, as well as some of the cis-acting structures located in the 5′UTR of gmRNA and sgmRNA that are relevant to this review (dashed circles). Viral replicase is involved in the synthesis of (−) ssRNA, (+) gmRNA and sgmRNA from dsRNA intermediaries (RF). gmRNA and sgmRNA are translated independently to produce nonstructural and structural proteins, respectively. Capsid protein recruits and assembles gmRNA to produce new virus particles. The accumulation of dsRNA molecules can trigger the activation of antiviral sensors (PKR, RIG-I and MDA5) that results in an antiviral response and the synthesis of type I IFN. As a countermeasure, viral nsp2 blocks transcription of the IFN gene in mammalian cells. Binding of a specific microRNA (miR-142-3p) to the 3′ NC region of EEEV that restricts the replication of this virus in hematopoietic cells is also shown [27].

The *Alphavirus* genus includes over 30 viral species that are classified in seven complexes based on their antigenic characteristics (ICTV Taxonomy 2022 release) (Figure 1A). Most alphaviruses are transmitted between vertebrate hosts (e.g., nonhuman primates, birds, rodents and marsupials) by hematophagous arthropods, mainly mosquitoes [1,2]. In general, the host tropism in alphaviruses is broad, and many zoonotic transmissions to humans have been documented. Thus, the chikungunya virus (CHIKV) has evolved from a sylvatic cycle between primates and forest mosquitoes to an urbanized transmission that involves mosquitoes of the *Aedes* genus (e.g., tiger mosquito) and humans [3,4,5]. The emergence of Venezuelan equine encephalitis virus (VEEV) strains causing infection in humans is also frequent [6]. Historically, mosquito-borne alphaviruses have been divided into Old World and New World viruses according not only to their geographical distribution, but also to the clinical manifestations they cause in humans [1]. Thus, Old World alphaviruses include the CHIKV, Sindbis virus (SINV), Semliki Forest virus (SFV), Ross River virus (RRV) and o’nyong-nyong virus (ONNV), which were first isolated in Africa and Australia and mainly cause arthritis. New World alphaviruses include the Venezuelan (VEEV), eastern (EEEV) and western (WEEV) equine encephalitis viruses, which are endemic to North, Central and South America and cause diseases in horses and humans with neurological symptoms [7].

Alphaviruses are enveloped, and their genomes consist of a positive-sense single-stranded RNA (ssRNA) of approximately 12 kilobases that encodes four nonstructural proteins (nsP1-4) and five structural proteins (capsid, E3, E2, 6k and E1) in two large open reading frames that are translated as polyproteins (Figure 1B) [8,9]. The nonstructural polyprotein is translated directly from the genomic RNA (gmRNA), whereas the structural polyprotein is translated from a subgenomic mRNA (sgmRNA) that is transcribed by the viral replicase from an internal promotor. Both gmRNA and sgmRNA are 5′ capped (m7Gppp or cap0) and 3′ polyadenylated (Figure 1B) [10]. The nsp1 has both N-7 methyltransferase and guanyltransferase activities that are responsible for the capping of a fraction of both the gmRNA and sgmRNA during RNA transcription [11,12,13]. The nonstructural protein 2 (nsp2) is a multifunctional protein involved in viral RNA synthesis, polyprotein processing, the blockade of host gene expression and viral pathogenesis [9,14]. In addition to its role as the main viral protease that cleaves the nonstructural polyprotein precursor (p1234), nsp2 also contains a NTPase/RNA helicase domain involved in viral RNA synthesis and a SAM (S-adenosyl-methionine)-dependent methyltransferase-like domain that cooperates with the RNA helicase domain [9]. The role of nsp3 in alphavirus replication involves the recruitment of multiple host factors towards the viral replication complex (vRCs), including members of the Ras–GAP SH3 domain-binding proteins family (G3BP) in mammals and the corresponding ortholog in mosquito (Rasputin) [15,16]. Interestingly, since G3BP is involved in stress granule formation upon stress-induced eIF2α phosphorylation, G3BPs hijacking by nsp3 has been proposed to induce the rapid disassembly of stress granules that are formed early in response to an infection, although very recent reports have questioned this idea [17,18,19]. Nsp4 is the viral RNA-dependent RNA polymerase involved in negative- and positive-stranded RNA synthesis to generate gmRNA and sgmRNA [20]. The regulation of these activities involves the sequential processing of the nsP1-4 precursor by the nsp2 protease [21]. In the early stages of infection, the partially processed nsPs (P123 + nsp4) preferentially synthesize a negative RNA strand to form double-stranded RNA intermediates (dsRNA). Later, the fully processed nsPs produce gmRNA and sgmRNA [21,22]. Recent data suggest that viral RNA synthesis initiates in the discrete foci associated with the plasma membrane to further translocate into the cytoplasm to form bigger membrane-bound spherules (or cytopathic vacuoles) containing the mature vRCs [23,24,25]. Although dsRNA replicative forms tend to accumulate into the spherules to serve as templates for the synthesis of new gmRNA molecules, the fact that antiviral sensors such as PKR, RIG-I and MDA5 become activated after alphavirus infection suggests that some viral dsRNA molecules are exposed to these antiviral sensors (Figure 1B) [26].

**Figure 2:**
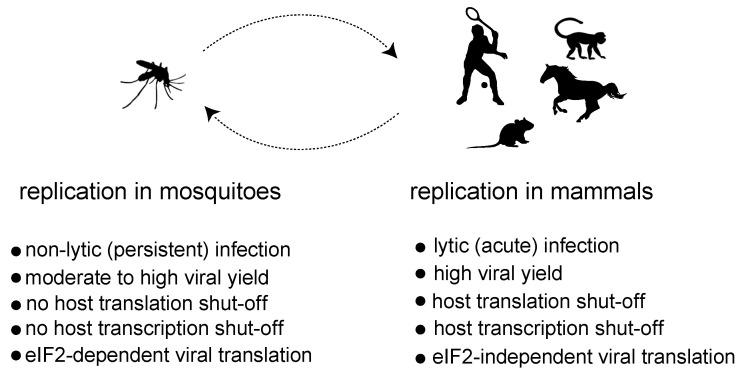
Comparison of the main characteristics associated with replication of alphaviruses in mosquitoes and mammalian hosts.

Although alphavirus replication has been studied mainly in mammalian cells, comparative analyses also found similar vRCs associated with the membrane-bound spherules in insect cells infected with SINV and other alphaviruses, suggesting that the basic aspects of alphavirus replication are conserved among these hosts [28,29]. However, the outcome of virus replication in mammalian and insect cells is different, suggesting the existence of underlying differences in the way virus replication impacts the host cell physiology. Alphavirus replication in mammalian cells is highly productive, and it is generally associated with a strong cytopathic effect that precedes cell lysis. In mosquito cells, however, alphaviruses easily establish nonlytic, persistent infections, where an active viral replication occurs without signs of a cytopathic effect (Figure 2) [28,30,31]. This fact nicely reflects how alphaviruses and other arboviruses use insects both as reservoirs to persist in nature and as vectors to ensure transmission to vertebrate hosts. At a molecular level, the replication of alphaviruses in mammalian cells is associated with a profound inhibition of both host transcription and translation that ultimately contributes to the cytopathogenesis. Thus, mammalian cells infected with representative Old World and New World members almost exclusively synthesize viral RNAs and proteins at later times of infection. In insect cells, on the contrary, the synthesis of large amounts of viral RNAs and proteins is well tolerated and occurs without significant interference with host transcription and translation [32,33]. This suggests that instead of relying solely on the replication process, alphaviruses employ specific mechanisms to halt gene expression in mammalian cells.

## 2. Mechanisms of Virus-Induced Host Translation Shutoff

Like many cytolytic viruses that infect mammals, an alphavirus infection often results in a strong blockade of host gene expression at both the transcriptional and translational levels [34]. The causative mechanisms of this interference or “shut-off” have been studied in both Old World (SINV and CHIKV) and New World (VEEV) members of the genus, using highly susceptible cell lines of human and murine origins [14,35,36,37]. Thus, the infection of BHK21 or murine embryonic fibroblasts (MEFs) with SINV and SFV at a high multiplicity of infection resulted in a complete shutoff of host translation at 3–4 h postinfection, concomitant with an almost exclusive translation of viral mRNAs, which can represent up to 30% of the total translation activity of uninfected cells. In other alphaviruses such as the VEEV and CHIKV, this effect is less dramatic in cell culture models [37,38,39,40]. Both the ongoing and de novo translation initiations of host mRNAs are abrogated in SINV-infected cells, suggesting that both the initiation and reinitiation (by ribosomal recycling) of mRNAs are blocked [35].

Different causative mechanisms have been proposed to explain the shutoff induced by alphaviruses [14,41,42]. Regarding mechanisms that impact the activities of translation factors, the strong phosphorylation of eIF2α by dsRNA-activated kinase (PKR) activation observed in cells infected with the SINV, SFV and VEEV could explain the translational block of the vast majority of mRNAs in infected cells [35,36,43]. eIF2α phosphorylation and host translation shutoff was also confirmed in mouse brain and organotypic cultures infected with the SINV [44]. However, SINV and CHIKV infections of PKR-knockout MEFs or PKR-knockdown human fibroblasts (HF), respectively, still induced a shutoff comparable to the control cells, suggesting the existence of an underlying mechanism(s) to halt host translation [35,36]. As discussed below, eIF2α phosphorylation in response to infection must be interpreted as an attempt of the host cell to prevent the translation of viral mRNAs in the context of an IFN response. More recently, a significant phosphorylation of elongation factor 2 (eEF2) has been detected in cells infected with many alphaviruses, including the CHIKV, SINV, SFV and VEEV [45]. eEF2 phosphorylation at the T56 residue reduces the activity of this factor, thus decreasing the rate of translation elongation [46]. The helicase activity associated with the NTPase domains of the CHIKV and VEEV nsp2s was sufficient to induce eEF2 phosphorylation and translation inhibition, although the contribution of this modification to the host shutoff induced by the CHIKV and other alphaviruses remains to be determined [45]. The activity of the nsp2s of Old World alphaviruses has also been linked to translation shutoff, since the SINV and CHIKV with point mutations in nsp2 that abrogated the host transcriptional inhibitory activity also failed to induce a complete inhibition of host translation in infected cells [14,36]. However, it is not clear if this defect in the translation shutoff could be an indirect consequence of a lesser impact of the mutated nsp2 on RNAPII activity that could make more mRNA available in the cytoplasm for translation. 

Other indirect causes of shutoff have been proposed, although the precise mechanisms involved and their contributions to the shutoff phenomenon are still an open question. Thus, the large quantities of subgenomic mRNAs (sgmRNAs) accumulated in cells infected with the SINV and SFV could sequester translation components, such as ribosomes and translation factors, to redirect translation towards sgmRNAs [35,47]. Although sgmRNA is efficiently translated in infected cells by acting as a super competitor mRNA, the fact that replicons of the SINV and VEEV, which lack the entire sgmRNA, still repressed host translation, which seriously limits the contribution of sgmRNA accumulation to the translation shutoff by these viruses [48,49]. Related to this fact, the viral RNA synthesis directed by alphaviral replicases has been correlated to host translation inhibition in SINV-infected cells. Thus, the addition of RNA synthesis inhibitors such as 6-aza-uridine or ribavirin reduced the accumulation of both genomic RNA (gmRNA) and sgmRNA in SINV-infected cells and partially prevented or delayed the inhibition of host translation [47]. Some authors also reported the release of nuclear proteins to the cytoplasm in SINV-infected cells that could be interfering with translation by sequestering host mRNAs [47,50]. However, a direct causative correlation between these observations and the shutoff phenomenon is still lacking, so their contribution remains to be measured. Early reports also described an inhibitory effect of the capsid protein of the SFV on host translation [51]. Finally, alterations in the plasma membrane permeability observed in cells infected with alphaviruses and other animal viruses have also been proposed as a cause of translation shutoff some decades ago, but currently, these lines of investigation have been discontinued.

## 3. Interferon-Induced Genes That Block Translation of Alphaviral mRNAs: Roles of IFIT-1, ZAP and PKR

Alphaviruses induce strong innate and adaptive responses that greatly influence the outcome of infection in animal models [7,52,53]. Thus, type I interferon (IFN-α/β) signaling is critical for the protection against many alphaviruses, in that mice devoid of type I IFN receptor (IFNAR) easily succumbed to infection by the SINV, SFV, CHIKV and VEEV [54,55,56]. In cultured cells, all alphaviruses tested to date showed a sensitivity to IFN-α/β pretreatment [40,57,58,59]. The infection of both primary and immortalized cell lines with alphaviruses may result in the secretion of significant amounts of IFN-α/β, and both RIG-I and MDA-5 sensors are required for an optimal response against SINV and VEEV [26,37,60,61,62]. However, in general, alphaviruses are not considered strong inducers of IFN secretion. As discussed below, the inhibitory effects of viral nsp2 and eIF2α phosphorylation on host transcription and translation, respectively, limit the synthesis of IFN and other antiviral cytokines in alphavirus-infected cells. 

IFN-α/β exert their antiviral effects through the activities of hundreds of genes whose expressions are stimulated by IFN signaling. Many of these interferon-stimulated genes (ISGs) encode proteins that are effectors of the antiviral response by blocking specific steps of the virus cycle such as decapsidation, RNA synthesis, viral translation or virus release [63]. Different combinations of these effectors can result in a specialized response against specific virus families, although some of them exert a broader antiviral activity [64]. Individual and combinatorial gene-targeting studies have identified a dozen ISGs that restrict alphavirus replication in cultured cells and in animal models [65,66]. Previous reports have shown that type I IFN pretreatment blocked the translation of alphaviruses, but not cellular mRNAs [67]. Interestingly, many of these IFN effectors exert their activity by blocking translation and/or by inducing the degradation of viral mRNAs, a finding that highlights the relevance of translation in controlling virus–host interactions and antiviral responses. These effectors include PKR, RNAseL/OAS, ISG20, ZAP, IFIT1 and IFIT3. In a recent loss-of-function screening analysis, the simple combination of three of these ISGs (ZAP, IFIT1 and IFIT3) accounted for the majority of the IFN-mediated restriction of VEEV and SINV replication in cultured cells [66].

**Figure 3:**
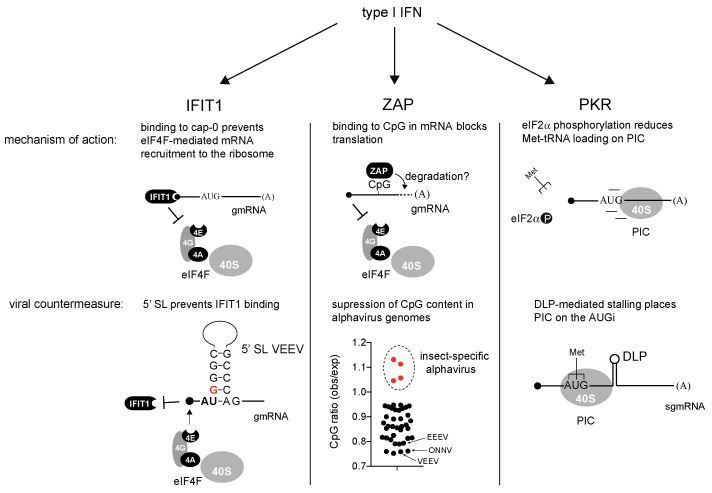
Mechanistic action of IFIT1, ZAP and PKR on the translation of alphaviral mRNA and some of the countermeasures used by alphaviruses to evade them. The 5′ stem loop in the gmRNA of VEEV (wt 3908 strain) is schematically shown. The G + 3, which is critical for IFIT1 resistance, is colored in red. To calculate the CpG ratio, the frequencies of CpG dinucleotides in full length genomes of 41 alphaviruses were divided by the product of corresponding C and G frequencies. Insect-specific alphaviruses are EILV, TALV, ASALV and MWAV. eIF2α phosphorylation not only prevents the location of AUGi of mRNA, but also reduces the formation of PIC (translation pre-initiation complex). For simplicity, the rest of components of the translation initiation complex were omitted. Black dots: alphaviruses infecting vertebrates. Red dots: insect-specific alphaviruses.

**IFIT1**. Interferon-induced proteins with tetratricopeptide repeats (IFITs) are robustly induced by IFN-α/β treatment. IFIT1 is the prototype of this family, showing the strongest antiviral activity against several virus families including *Alphavirus* and *Coronavirus* [68,69]. The antiviral effects of IFITs depend on their ability to bind the 5′ extreme of mRNA to block the translation initiation [70]. However, the presence and type of cap structure greatly influences the affinity of the IFIT proteins for mRNAs. IFIT1 can interact with cap0-mRNAs, the simplest version of the cap structure, whereas IFI1B can also interact with cap1-mRNAs that contain an additional 2′-O-methylation at the first nucleotide of the mRNA [71]. Some groups also reported the interaction of IFIT1 with uncapped 5′-ppp-mRNA, although further studies suggested that only IFIT5 was able to interact with 5′-ppp-RNA in vitro [72]. In addition, IFIT3 can modulate the cap-binding affinity/specificity of IFIT1 [73,74]. Interestingly, further 2′-O-methylation at the second nucleotide of the mRNA (cap2) completely prevented the interaction of IFI1/1B with the cap structure, allowing the cellular mRNAs that generally contain cap2 structures to escape from IFIT-mediated inhibition [72]. Alphaviruses, like other viral families, encode their own capping enzyme (nsp1), but they lack 2′-O-methyl activity, so the resulting cap0-mRNAs are targets of IFIT1-mediated translation inhibition (Figure 3). Elegant experiments using chimeric nonreplicative alphaviruses showed that IFIT1 blocked the translation of incoming gmRNA upon IFN treatment [75]. However, as discussed below, not all the alphaviruses are equally sensitive to IFIT1 because of adaptive changes to evade the IFN response. 

**ZAP**. The zinc finger antiviral protein (ZAP or ZC3HAV1) is able to inhibit the replication of many alphaviruses including the SINV, RRV, SFV and to a lesser extent, the VEEV, CHIKV and ONNV, as well members of other RNA and DNA virus families [65,76,77,78,79]. The ZAP is expressed as four splice isoforms that share an N-terminal domain with four CCH zinc fingers that are directly involved in RNA binding [80,81,82,83]. The large isoform (ZAPL) is constitutively expressed, thus providing the cells with basal ZAP activity, whereas the small isoform (ZAPS) is significantly induced by type I IFN treatment (2–3-fold increase) [81,82]. ZAP binding to viral mRNAs restricts virus replication by blocking translation and/or inducing the degradation of the target mRNAs (Figure 3) [77,84]. Structural and biochemical analyses showed that the RNA binding domains of mammalian ZAPs bind ssRNA molecules containing CpG dinucleotides with a high affinity [85,86,87]. This preference for CpG-rich regions in RNAs has been interpreted as the basis for ZAP specificity that could discriminate between self (host) and nonself (viral) mRNAs, since CpG is generally underrepresented in mammalian genomes [85,88]. Although ZAP binding to specific regions of the HIV-1 and SINV that are rich in CpG dinucleotides has been reported, the basis for this specificity has not yet been fully defined [80,87,89]. ZAP binding requires not only the proper spacing of CpGs, but also, the nucleotide composition surrounding CpGs influences the ZAP’s affinity for target RNAs [86,90]. In alphaviruses, the ZAP blocked the translation initiation by preventing the assembly of the cap binding complex (eIF4F) on the incoming viral gmRNAs at early times of infection (Figure 3) [84,91]. However, since the ZAP preferentially binds to internal regions of the SINV gmRNA, it is not clear how ZAP binding prevents mRNA recruitment by the eIF4F complex, which occurs distally [80]. The destabilization of alphaviral mRNAs upon ZAP binding could also occur as described in other viruses, so that both mechanisms could be operating to restrict the replication of alphaviruses [92]. For alphaviruses, the longer ZAP isoforms showed a better antiviral effect [82]. A full ZAP activity requires cofactors such as the ubiquitin E3 ligase tripartite motif-containing protein (TRIM25) that also interacts with viral mRNAs [91,93].

**PKR**. The dsRNA-activated protein kinase (PKR) was the first ISG described, and it represents one of the four eIF2α kinases that respond to stress in mammals [94,95,96,97]. Although constitutive levels of PKR are present in most cell types, especially in fibroblasts, IFN treatment increases up to 10-fold the amount of PKR in the cell [95,98]. PKR is one of the main effectors of IFN-α/β, and animals devoid of PKR are much more susceptible to RNA and DNA viruses [97,98,99]. PKR plays a dual role as a sensor and IFN effector, owing to the presence of two dsRNA binding motifs (dsRBMs) at the N-terminal of the kinase [96]. The accumulation of dsRNA molecules as byproducts of viral RNA replication activates the PKR that phosphorylates the eIF2α at the S51 residue, thus preventing the recycling of this factor necessary to charge 40S subunits with new molecules of Met-tRNAi for translation initiation (Figure 3) [95,96]. Even low levels of eIF2α phosphorylation in response to PKR activation can have an amplified impact on translation, because phospho-eIF2 acts as a competitive inhibitor of eIF2 recycling, which involves the GTP-exchange factor eIF2B [100,101]. Since eIF2α phosphorylation leads to a general blockade of both viral and host translations, the effect of PKR activation can be considered less discriminative when compared to ZAP and IFIT1. Most pathogenic viruses express viral antagonists that prevent or counteract PKR activation in infected cells, thus making these viruses more resistant to IFN [96,102]. However, alphaviruses generally do not prevent the activation of dsRNA-activated kinase (PKR) in infected cells [35]. In return, they have acquired the ability to initiate translation independently of eIF2 as discussed below.

## 4. Adaptive Changes in Alphavirus mRNAs to Counteract the Antiviral Effect of IFN

As a result of thousands of years of virus–host coevolution, most pathogenic viruses adapted to counteract the antiviral activity of IFN [103]. In alphaviruses, these adaptive changes involved the acquisition of cis-acting elements in genomic and subgenomic mRNAs, changes in the dinucleotide composition and the occurrence of new activities in viral proteins that greatly influence viral translation and host gene expression. Collectively, these features and activities are aimed to promote the resistance to IFN (Figure 3). 

**IFIT1 resistance.** Pioneer work by Hyde et al. reported a single mutation (G to A) at nucleotide 3 in the 5′ UTR of the gmRNA of the VEEV that drastically increased the sensitivity to IFIT1 and IFN in cell culture and animal models [69]. A structural analysis confirmed the existence of a short stem–loop located at the 5′ extreme of gmRNA, whose stability (and/or topology) reduced the binding affinity of IFIT1 for VEEV gmRNA (Figure 3). Further studies found equivalent RNA structures in other alphaviruses, including the SINV, SFV, CHIKV and EEEV, that also promoted the resistance to IFIT1 at different extents depending on the virus [75]. A mutational analysis showed that, to generate IFIT1 resistance, the two first nucleotides of the gmRNA need to be AU (paired or not) and need to be followed by a G–C-rich stem likely involved in structure stabilization and/or topology (Figure 3) [75]. Interestingly, the existence of this structure was originally described in alphaviruses as a conserved element (CSE 1) acting as a promotor for the synthesis of both negative and positive RNA strands [8]. This raises the possibility that a previous structural element in the 5′ extreme involved in virus replication could also be adapted to shield the gmRNA against IFIT1, thus mimicking the protective effect of 2′ methylation at the first and second positions in cellular mRNA [69]. However, not all pathogenic alphaviruses are equally resistant to the antiviral effect of IFIT1. Thus, the EEEV shows only a partial resistance to IFIT1 that is probably compensated by the low levels of type I IFN induced by this virus in cultured cells and animals [53,75,104].

**ZAP resistance**. As described above, ZAP binds CpG-rich regions in mRNA to promote the translational repression and further degradation of alphaviral gmRNAs. However, since alphaviruses are not equally sensitive to ZAP-mediated restriction, some reports have analyzed the correlation between the CpG content of viral genome and the ZAP sensitivity. At the genomic scale, no obvious correlation was found between the sensitivity to the ZAP and the CpG composition among the main pathogenic alphaviruses analyzed. Therefore, some reports suggested that alphavirus evasion of the ZAP correlated with CpG suppression in specific regions of gmRNA, although this possibility remains to be confirmed [89]. Expanding the focus to include the insect-specific alphaviruses in the analysis could shed light on the phenomenon of CpG suppression in pathogenic alphaviruses. Insect-specific alphaviruses includes EILAT (EILV), TaiForest (TALV), AguaSalud (ASALV) and Mwinilunga (MWAV), which are close to the WEEV group, but they are unable to replicate in vertebrate cells [105,106,107,108]. The CpG frequency found in these viruses fits what is expected according to the C and G compositions of their genomes (Figure 3). In contrast, a clear overall CpG suppression was found in the rest of the vertebrate-adapted members of the genus, especially in the VEEV and ONNV, which showed the lowest sensitivity to the ZAP (Figure 3) [75]. Since no orthologs of the ZAP and IFN genes are found in insects, insect-specific alphaviruses have not been subjected to the selective pressures imposed by these genes, so that CpG suppression found in the rest of the alphaviruses could be interpreted as a footprint of ZAP evasion, as described previously for other viruses [85,109]. Then, a further suppression of the CpG content in specific regions of the viral genome targeted by the ZAP could explain the reduction in ZAP sensitivity found in some pathogenic members of the genus such as the VEEV and ONNV. Alternatively, viral resistance to the ZAP might also involve changes in the nucleotide sequences around the CpG, which are still to be deciphered, that could be modulating the ZAP affinity for target RNAs.

**PKR resistance.** Many Old World alphaviruses have acquired cis-acting RNA structures in sgmRNA to counteract PKR-mediated αα phosphorylation in infected cells [110]. Genetic, functional and structural analyses have shown the existence of a stable stem–loop structure (DLP) located 27–30 nt downstream the AUG of sgmRNA in the SINV and SFV that confer resistance to PKR-mediated eIF2α phosphorylation (Figure 3) [35,110,111]. A functional analysis also showed the existence of equivalent structures in the RRV, Sagiyama virus (SAGV), Bebaru virus (BEBV), Una virus (UNAV), Middelburg virus (MIDV) and to a lesser extent, in the EEEV [110]. The DLP activity greatly depends on the RNA structure stability, especially at the base of the stem, whereas the helix topology seems to be less relevant. Thus, structure destabilization by introducing adenines (A) in the C–G-rich stretch of the stem abolished DLP activity [35]. The distance of the DLP to AUGi was found to be critical, so the DLP structure must be located in a 24–37 nt window downstream the AUG of the sgmRNA to promote resistance to eIF2α phosphorylation [110]. Crosslinking experiments of 48S-PIC assembled with the SINV and SFV sgmRNAs revealed multiple contacts of DLP with nucleotides of 18S rRNA in the ES6S region, suggesting the existence of a trapping mechanism that promotes 48S-PIC stalling in a way that allows the proper placement of AUGi in the P site during the scanning process (Figure 3) [110]. Thus, the DLP-mediated stalling of a translation initiation complex would increase the resistance to PKR by reducing the dependence of sgmRNA for eIF2 in mammalian cells, or by allowing an eIF2-independent mechanism for the recruitment of Met-tRNA. Interestingly, the SINV mutant lacking a functional DLP, or aura virus (AURAV) that naturally carries a suboptimal DLP structure located too close to the AUGi, both showed very low levels of replication in primary cells and mice, and a dramatic increase in their sensitivity to IFN in vitro [59]. Moreover, an SINV mutant lacking DLP rapidly evolved after serial passages in MEFs to partially restore the DLP structure, a finding that stresses the importance of the DLP structure in alphavirus replication and PKR resistance [32]. However, in other alphaviruses such as the CHIKV, ONNV and Mayaro virus (MAYV), the presence of this structure in the sgmRNA was not detected, suggesting that the acquisition of DLP was not a universal trait in alphavirus evolution. Interestingly, the productive infection of cultured cells with the CHIKV resulted in very low levels of eIF2α phosphorylation as compared to the SINV or SFV [19]. This suggests the existence of an alternative strategy in this virus to prevent eIF2α phosphorylation, perhaps by the presence of a viral protein with an anti-PKR activity as described in many other virus families [112].

**Suppression of IFN synthesis by nsp2s and capsid proteins.** The activities of Old World nsp2s and New World capsid proteins have been linked to the transcriptional shutoff observed in vertebrate cells as an effective mechanism to prevent the synthesis of IFN and other antiviral cytokines by pathogenic alphaviruses. In addition to their function in replication and virus encapsidation, a significant fraction of Old World nsp2s and New World capsid proteins accumulate in the nuclei of infected cells at early times of infection, where they suppress host transcription and/or nuclear trafficking [113,114,115].

An extensive genetic analysis has firmly established that the Old World nsp2 blocks type I IFN transcription by inducing the proteasomal degradation of Rpb1, a catalytic subunit of the RNAPII complex [33]. This prevents the expression of IFN and other antiviral genes in response to infection, highlighting the role of nsp2 in innate immune evasion [60,116]. Rpb1 degradation involves polyubiquitination and depends on the integrity of the helicase and SAM-dependent methyltransferase-like domains of nsp2, so that point mutation in these domains can abolish Rpb1 degradation and lead to the subsequent halt of host mRNA synthesis [33,116]. Consequently, the SINV and CHIKV viruses carrying some of these mutations in nsp2 became potent inducers of type I IFN in primary cell cultures that resulted in a marked attenuation of virus replication, especially in vivo [116]. Importantly, these mutant viruses also lost their pathogenic potential when inoculated in mice, highlighting the relevance of nsp2 activity in virus–host interactions [37,116].

Surprisingly, the transcriptional shutoff induced by New World alphaviruses is carried out by a capsid protein rather than by nsp2. However, contrary to nsp2, the VEEV capsid protein did not induce the degradation of any RNAPII subunit [113,114]. The expression of the VEEV capsid protein both individually, and in the context of viral infection, potently blocked the host transcription and nuclear import in mammalian but not in mosquito cells [115]. A short region located at the N-terminals of the capsid proteins of VEEV and EEEV promotes the formation of a tetrameric complex of the capsid protein with CRM1 and alpha/beta-importin that obstructs the nuclear pore, thus blocking the nucleocytoplasmic transport [117]. Moreover, the N-terminal region of the capsid protein is essential for the transcription suppression activity of the VEEV capsid protein, suggesting that the blockade of nucleocytoplasmic transport could be indirectly responsible for transcriptional suppression. Although this was further supported by the observation that the blockade of nuclear import precedes the transcriptional shutoff, a causal relationship between these two phenomena remains to be established.

The occurrence of transcriptional suppression activities in different proteins of Old World and New World alphaviruses constitutes a remarkable case of evolutionary convergence that stresses the central role of IFN evasion mechanisms in the natural story of alphaviruses in their two main geographic areas of distribution. 

## 5. Conclusions and Future Directions

The interference of alphaviruses with host translation seems to be a multifactorial process that results from the intricate balance between host-induced measures and virus-induced countermeasures. The interplay between alphavirus-induced transcription and translation shutoff deserves to be explored in the future, as well as the impact that the cytoplasmic accumulation of many RNA binding proteins observed in infected cells could have on host translation. To what extent IFN evasion mechanisms have shaped the genome of alphavirus also deserves further investigation to obtain clues on some aspects of the origin and evolutionary history of alphavirus that still remain controversial. This will require a better understanding of ZAP specificity that will allow us to predict the existence of ZAP binding sites in viral genomes. 

## Data Availability

No specific data were generated to support reported results.
